# Factors Associated with Successful Mentoring of Parents Addressing Childhood Obesity: A Mixed Methods Approach

**DOI:** 10.1155/2016/5769621

**Published:** 2016-11-20

**Authors:** Gabriela Abigail Villanueva, Byron Alexander Foster

**Affiliations:** ^1^Department of Obstetrics and Gynecology, Texas Tech University Health Sciences Center, El Paso, TX, USA; ^2^Department of Pediatrics, University of Texas Health Science Center at San Antonio, San Antonio, TX, USA

## Abstract

*Objective*. Parents mentoring other parents as a behavioral intervention for child obesity is novel with limited data describing the experience and dynamics of this approach. This study aimed to describe the experiences of parent mentors and the self-efficacy and attitudes of their mentees in the context of a clinical trial for childhood obesity.* Methods*. The context for this study was a randomized clinical trial using either parent mentors or a community health worker engaging parents of obese children in behavioral change over six months. Parent mentors were interviewed at the mid-point of the intervention using a semistructured questionnaire to elicit their perceptions and experiences during the process of mentoring. Parent mentees completed a survey assessing their self-efficacy, perception of the parent mentor, and attitudes and beliefs related to their child's weight.* Results*. The qualitative analysis of parent mentor interviews indicated high commitment despite their nonprofessional status, facing challenges of engagement with fellow parents and attitudes of persistence and being nonjudgmental. The parent mentee ratings of parent mentors were overall very high and similar to the ratings of a community health worker (paraprofessional).* Conclusion*. The data suggest that a parent mentor model of intervention for child obesity is an acceptable mode of approaching behavior change in the Hispanic population around childhood obesity with potential for scalability if proven effective.

## 1. Introduction

Parent mentors have been used in interventions aimed at improving childhood asthma control [1], reducing malnutrition [2], and improving general parenting skills [3]. They have also been used to provide support for families of children with chronic disabling conditions [4]. New research has shown that using parents may be of greater benefit because of their ability to relate to the experiences of other parents [5]. There are limited data describing the dynamics of the mentoring relationship when parent mentors are being used in an interventional rather than supportive context and how they are perceived by their mentees. This is an important gap given the heterogeneity in these types of interventions and the need to better contextualize outcomes between studies.

There are few studies that have used a qualitative approach to assess the experience, behaviors, and activities of mentors [6–8]. One study found a strong role for affirmational and emotional support between mother mentors and their mentees and less emphasis on providing information or discussing specific medical information [7]. Increased self-efficacy and empowerment have been described in parent mentors, with one report describing the mentors' job opportunities improving postmentoring [8]. Other investigators found that mentors are able to empathize with participants and provide affirmational support [9, 10].

The current report examines the experiences of parent mentors involved in a child obesity intervention study in a Hispanic population using primarily a qualitative approach with complementary quantitative data. Feeding and disciplinary behaviors are deeply rooted in culture and family and often intensely personal. Furthermore, obesity in early childhood among the Hispanic community is often not recognized as a problem. Therefore, the challenges that parent mentors face in this context, despite coming from the same community, may be different from prior applications of the parent mentor model. Mentors may be faced with challenging closely held family beliefs about the provision of food for comfort or suggest changes for the child that go against the normative behavior for the family's social context.

This project involved a clinical trial enrolling obese, 2–5-year-old children and their parents (dyads). The original study aimed to compare differences in parent-child dyads enrolled in the study that were assigned to either a parent mentor or a community health worker, both of whom delivered education and behavioral coaching over the course of 6 months as described previously [11]. This paper aims to better understand the experiences and perceptions of parent mentors and how this translates to the experiences of their mentees. A better understanding of this dynamic could lead to greater success in future interventions.

## 2. Methods

### 2.1. Overall Study Design

The overall study was designed as a randomized trial targeting Hispanic, obese, 2–5-year-old children and their parents who were enrolled in a Head Start program (a federally-funded early childhood education program for low-income families) in the lower Rio Grande Valley in south Texas [11, 12]. There were 60 parent-child dyads enrolled in the study, and they were randomized 1 : 1 to receive teaching from either a parent mentor or a community health worker using a standardized curriculum [13].

The parent mentors were all mothers who were identified by the Head Start staff as having positive personalities and potential leadership qualities from among the other parents enrolled in the Head Start program; while fathers were potentially eligible to participate, none volunteered. They were also selected for being bilingual in English and Spanish. The mentors otherwise had no prior mentoring experience and were selected based on a brief, unstructured interview.

Parent-child dyads in the parent mentor arm of the trial were paired with a trained parent mentor. Parent mentors were trained in a one-day session using a bilingual handbook developed with input from the pilot study on positive deviance methods derived using local parental input, as previously described [11]. The theoretical framework of positive deviance drove the development of the handbook and the overall intervention design. Positive deviance is a complexity-theory based model which asserts that, even among those most at risk for a poor outcome, there are some individuals who find ways to succeed, and by learning from their success, we may be able to discover innovative and sustainable means of addressing the problem in the rest of the community at risk.

Parent mentors were instructed in active listening, reflective feedback, and peer coaching techniques as well as the positive deviance findings and local resources for parents of obese children. The parent mentors conducted a baseline home assessment with the parent and child and conducted follow-up phone calls at least once a month to encourage healthy habits and behaviors identified as foci for that particular parent. The parent mentors were given complete flexibility in terms of how often they contacted their mentees, what topics they chose to focus on, and how they interacted with the families. The parents were invited to participate in monthly community meetings facilitated by the parent mentor and focused on encouraging the behaviors previously identified as positively deviant and healthy habits identified as goals by the group. Each of the three parent mentors had 10 families assigned to them.

Parent-child dyads randomized to the community health worker condition were also invited to attend community meetings on a monthly basis at Head Start centers. These meetings were facilitated by a local* promotora*, or community health worker, who used the EatPlayGrow™ curriculum to teach about healthy habits. The EatPlayGrow curriculum uses an interactive format to engage parents and children, and it has been piloted in multiple early childhood settings among diverse populations [13]. Parent-child dyads in the community health worker condition did not receive a home visit nor any follow-up phone calls.

### 2.2. Qualitative Methods

We interviewed all three of the parent mentors involved in the current study. They were all Hispanic mothers of at least one child 2–5 years of age who was at a healthy weight. The interviews were recorded using a digital voice recorder and then professionally transcribed with Adrianna, Bianca, and Cynthia used as pseudonyms.

We conducted the interviews in March 2015, three months into the overall study which began enrolling January 5, 2015. We interviewed each parent mentor once, using the same questionnaire. The interview guide was semistructured and flexible to allow for the interview to take its own direction. A junior staff person involved in the research project conducted the interviews. The basic outline for the interviews is available as a supplemental file (Supplemental File 1 in Supplementary Material available online at http://dx.doi.org/10.1155/2016/5769621).

### 2.3. Quantitative Methods

#### 2.3.1. Survey

We also sought to understand whether the parent mentees participating in the study perceived the parent mentors differently than the more traditional community health worker. We administered a survey consisting of questions on perceptions of the parent mentors or community health worker, self-efficacy, and attitudes related to childhood obesity. Likert-scale-based questions that prompted the parent to rate their self-confidence in addressing childhood obesity were also included. The parents' self-efficacy was assessed using the General Self-Efficacy Scale, a 10-item, Likert-scale questionnaire [14].

The questionnaires were first written in English and then translated into Spanish. The translated version as well as the English version was piloted by bilingual volunteers. The General Self-Efficacy Scale Spanish version was used instead of being translated, as it has demonstrated to be valid and a reliable measure among the Spanish speaking population in clinical and community settings [15]. Surveys were administered at the end of the study (after six months of intervention).

### 2.4. Ethics and Incentives

This study was approved by the University of Texas Health Science Center at San Antonio Institutional Review Board. Both parent mentors and parents of obese subjects provided written consent to participate. All subjects were provided with an incentive to participate as previously described [11].

### 2.5. Analysis

#### 2.5.1. Qualitative Analysis

We used qualitative description [16] to stay as close to the data as possible, in part given the limited prior research. Given the pilot and exploratory nature of this study, we focused on describing the experiences, successes, barriers, and challenges faced by parent mentors. Validation was done during the interviews to elicit immediate feedback. Member checking was done with the mentors on the broad themes identified during a debriefing session two months after the end of the mentoring period.

The interviews were read at least three times to get an overall sense for the flow and tone of each interview. Each interview was separately coded, using the web-based Dedoose application. Coding was done once for each interview by both emic and etic coding. After all interviews had been coded, each code was reviewed within each individual interview again; some codes were modified in this second round. For the third round, codes were again reviewed, both within each interview and across interviews looking for commonalities and emergent themes.

#### 2.5.2. Quantitative Analysis

We used descriptive statistics to analyze the survey data administered to the parents enrolled in the study, the parent mentors, and the community health workers. Data were collected using REDCap [17] and then transferred to SPSS 23.0, IBM (USA), for descriptive statistical analysis.

## 3. Results

### 3.1. Qualitative Results

All three mentors were in their early 30s. Adrianna was enrolled in a technical-college, her husband was unemployed, and they have one young child (age 2–5), and she was obese herself. Bianca was unemployed, her husband is deceased, she has one young child (age 2–5) and two older preteen boys, and she was at normal weight. Cynthia was employed part-time in hospice care and has four children with the youngest aged 2–5, her husband works part-time, and she was obese.

Four overarching themes emerged from the data: perceived outcomes of participation, parent mentor qualities, obstacles and resources to overcome, and building community. While the themes are discussed separately, we recognize that they are intertwined within each individual, and the dynamics between the mentor and participants is an evolving process. For example, the parent mentor qualities described clearly inform the obstacles they faced and the ways they approached them. Their individual life experiences and qualities influence how they perceive the outcomes of participation and how they are each engaged in building community.

#### 3.1.1. Perceived Outcomes of Participation

Each mentor described in some way how their participation in the study had affected their own life or habits. Adrianna, who had the highest level of education of any of the mentors, seemed pleasantly surprised that she had learned from the process. Bianca described concrete ways in which being a mentor had affected her.

*“Well, it's changed my shopping experience for sure with my children. I don't give them snacks anymore. I try to stay away from the sweets. I myself have been changing with my children, so it's helped me a lot.”*

Cynthia discussed how her mentoring had an effect on her as she described implementing what she was teaching in her own life. While she had a child who was at a normal weight, her awareness of the process and challenges of healthful eating seemed heightened.

In discussing how the study had influenced the participants they were mentoring, the mentors discussed process change or the beginnings of change.

*“When we're talking in conversations they will actually open up and say, ‘Yeah, well, you know what? I fried this but you know what? I'm not doing that as often' or ‘You were right about some points in the meeting and we're going to try this.' It's been a really positive experience.”*

Interestingly, none of the mentors discussed weight or appearance of weight in their perceptions of success or effects of the study. Also, they tied their own experience to the description of the mentee's experience.

#### 3.1.2. Parent Mentor Qualities

While each of the parent mentors had a unique personality, commonalities did emerge. Being nonjudgmental was described explicitly by each mentor.

*“It's just letting them know that I am just there to help them, I am available and I'm not there to judge, most important because I think in the beginning, when somebody comes and tries to change or tell you that you're making them feel they're not doing something, they might be a little bit more defensive.”*

The tone and context of their descriptions implied that they were either reflecting on how some of the mentees in the study initially perceived their possible role or how they themselves might have perceived being mentored on their child's health.

The mentors seemed to have significant commitment to their role. It should be noted that this was despite significant resource limitations: one of the parent mentors (Bianca) had no car and the other two had other significant commitments (Adrianna in school and Cynthia with work) in addition to being primary caregivers for their own children. Adrianna described the intervention as something she owned rather than being a study subject.

*“I mean, I do care about my parents. I want this, a study on my part, to succeed. I am giving it my best and I'm giving it my all. I want them to feel that I do care. That we're trying to do something positive and it's not just a temporary get to know you and then goodbye thing.”*

Another common attribute was being encouraging. Adrianna described being encouraged in contrast to what may be expected, even contrasting with what may be commonly said or heard at a health professional's office.

*“I'm able to break it down to a certain level where it's just like a friend to friend talk, versus a medical doctor with big scientific words that as soon as you go home you have to look in a dictionary in order to understand what he just said… I believe that there's a difference and it feels, to me, that it might be a little bit better from coming from a parent mentor.”*

Cynthia described focusing on making little changes and focusing on the positive.

#### 3.1.3. Obstacles and Ways to Overcome

When asked to discuss any obstacles or challenges, the parent mentors primarily discussed engagement with the parent mentees. The parent mentors were being potentially put outside of their usual social circles and asked to provide advice and coaching. Another challenge was addressing the individual backgrounds, experience, and time constraints of their mentees. Bianca had the least education of the mentors, and she vividly described being intimidated by her own perceived lack of expertise or knowledge.

*“The first time that I met with them it's like I was learning from them, but then again I have to teach them that this is a child and what portions to feed him and of course stay away from the sweets because they had a little issue with wanting cookies and ice cream. They've been doing a lot better on that themselves before I even met them. I think that was a little challenging for me. I didn't know what to tell them to help them because they already knew.”*

Cynthia also described a unique challenge in which the mother who was her mentee was enrolled in the program (voluntarily) but, at the same time, really did not seem to want to engage.

In accessing and using resources to mentor their parents, each mentor described a slightly different strategy. Cynthia in particular was very careful about the resources she chose, mostly the booklet she was given during training and making sure her sources were reputable, perhaps reflecting her own experience working in hospice. Somewhat in contrast and perhaps reflecting her higher educational background, Adrianna described her use of independent resources that she sought out, primarily government, health, or education websites and scholarly journals. All the mentors described the challenge of time. Tied into the notion of ownership described above, Adrianna described her approach to that challenge.

*“…we're busy, they're busy and it's challenging just to kind of meet in the middle. You will have some parents where they'll really, really try and they'll try and they'll try but they're just… They're busy and you're just going to really have to put more effort and more work to meet, especially to meet the demands, meet your duties that you're supposed to do within a certain time.”*

#### 3.1.4. Building Community

One of the unexpected results of the project was the connection between the parent mentors and their mentees described early on in the study. Each mentor described sharing, openness, and friendliness around the study that seemed to transcend the structure of the context of a randomized controlled trial. Cynthia's description of her role was striking as a focal point for the beginnings of a community, describing openness and friendliness.

*“Everybody seems to be friendly with me. They seem open and some of them want to introduce me to some of their friends so that I get to all their friends about what they're learning and I said, ‘No, that's where you come in. You teach your friends.' That's where the chain goes.”*

### 3.2. Survey Results

Forty-six of the forty-eight participants who completed the overall study completed the end of study survey. The parent mentees rated the parent mentors on their abilities in the community meetings similarly to the paraprofessional community health worker (Table 1). The only difference between the two groups was that the parent mentor group reported a higher ability to identify with the presenter (*p* = 0.04). Finally, the lowest rated item was whether obesity was a significant problem in the community with an overall mean of 79, SD = 29.

There was no difference in self-efficacy between the parent mentor group and the community health worker group with both having a high level of self-efficacy (median = 37, IQR = 5, *p* = 0.47 for differences between groups using Mann-Whitney *U* test). The mentees were divided into two groups based on self-efficacy score: those who fell above versus below the median (median = 37). We examined whether these levels of self-efficacy were associated with attitudes toward behavioral change. Those scoring above the median on the self-efficacy scale reported feeling more confident finding resources (*p* = 0.05) and higher confidence in making healthier choices for their children (*p* = 0.06) though both parents reported mean confidence scores >90 both above and below the median self-efficacy groups. Interestingly, there was also a moderate correlation (0.42, *p* = 0.003, Spearman's coefficient) between self-efficacy scaled on a continuum and reported time spent with their parent mentor.

## 4. Discussion

Parent mentors in this study on childhood obesity described an overall positive impact of participation on both themselves and perceived a positive impact for their mentees; they described using a nonjudgmental and supportive approach and formed significant bonds over a relatively short period of time. This model of using parents as a primary intervention for behavior change in childhood obesity has theoretical appeal since there are multiple existing structures where this could be implemented (schools, Head Start, daycares, etc.). Leveraging the people in the community to promote positive habits has significant appeal for a public health problem as large as childhood obesity. While the efficacy of these parent mentors in changing behaviors or reducing adiposity is outside the scope of this paper, the data discussed here support the feasibility and cultural acceptability of this model which is an important outcome of its own which has implications for similar studies.

One of the clear themes that came across was the nonjudgmental attitude, openness, and even friendliness described in the dynamic between mentor and mentee. The literature describes some of these same principles on how to form a therapeutic alliance between doctors and patients around obesity [18]; these parent mentors were able to achieve such an alliance in a short amount of time. The parent mentors also described having high self-efficacy related to their experiences, which has been noted in another parent mentor study [8]. The parent mentors also openly described some of the personality conflicts they encountered and the pressures of time; they seemingly dealt with these conflicts and negotiated these pressures very effectively. Given the fairly low level of support provided by study staff, this speaks to their character prior to the study rather than the training component. We conducted individual interviews with potential parent mentors after they were selected by their peers. In expanding this model of intervention, developing a more streamlined process for mentor selection, recruitment, and training should be considered.

Parental beliefs and attitudes are a reflection of both the community and what was being taught by the peer mentors. The rating for believing there was a problem of childhood obesity was lower than expected and rated lower than any other question. This is consistent with a comment attributed to a parent by one of the parent mentors: “I don't think my son needs this… I don't think he's chubby. I think he's underweight.” None of the parent mentors ever mentioned trying to prove to parents that there was in fact a childhood obesity problem; indeed the subtheme of being nonjudgmental may reflect an underlying belief in the community at large. Recognizing that childhood obesity is a problem has been shown to be a barrier to modifying behaviors among Hispanics in several studies [19]; however, having a mentor that does not focus on what parents have done wrong may be beneficial. A parent mentor from the community may also have the advantage of better identifying with the parent in helping them recognize the aspects of their life they can change.

Most parents rated high confidence in being able to find resources regarding childhood obesity. The peer mentors themselves mention several resources that they used for information: “remind them, they can ask their pediatrician, they can ask a dietitian at school, they can ask a teacher…” and so it is possible that these reminders may have helped the parents feel that they can find resources themselves. It is well documented that Hispanic mothers have miscued perceptions of feeding and the association of physical activity, nutrition, and health; therefore, feeling confident that they can find reliable resources could significantly improve knowledge of feeding and obesity [20, 21]. Parents who scored above the median in self-efficacy were more likely to feel confident in making healthier choices and feeling that their child could achieve a healthy weight. This reflects the general principle of self-efficacy: overcoming barriers even in the state of adversity. This is important as all of these parents are from low socioeconomic backgrounds and face barriers such as being able to afford healthy food and going against cultural practices. Among Caucasian white females in a health promotion intervention for weight loss, general self-efficacy scores among those with a higher BMI were found to be on average 31.5 [22]. A study using Latina migrant farmworker mothers in a health promotion intervention reported a general self-efficacy score among the Latina mothers to be 30.3 [23]. Our population reported much higher scores at the end of the intervention, with a median of 37. No difference was found between the community health worker group and the parent mentor group. The internal consistency of the data is reinforced by the finding that those above the self-efficacy median even despite the overall high scores were more likely to report higher confidence in making a behavioral change such as making healthier choices, finding resources, and believing that their child can achieve a healthy weight [24].

This is the first description to our knowledge of the experience of parent mentors working with other parents to reduce their child's obesity. While there were some challenges to implementing a parent mentor model of behavior change for obesity, the parent mentors were able to overcome those challenges and had an overall very positive experience that has potential for replication and dissemination to address this significant public health problem.

### 4.1. Limitations

There were several limitations to this study. All of our mentors were females chosen by the staff and showing an interest in participating; while the study could have included fathers as mentors, none volunteered or were chosen. In other studies, mothers have been shown to play an integral role in controlling nutrition in Hispanic households, and it has been demonstrated that they are an important component in preventing obesity in their children [25, 26]. Fathers were not excluded in this study and several did attend the education classes with their spouses. Further research to examine roles of fathers and mothers in this type of intervention may be warranted.

The two interventions of a community health worker versus a parent mentor also differed in not only the type of interaction and content but also the intensity of time. The parent mentors spent one-on-one time with their mentees either in person or over the phone providing individual support and advice. The lack of an attentional control group in this study is a limitation in making any comparison between the groups.

## Supplementary Material

Supplemental file 1 contains the basic outline for the semi-structured interview conducted with each parent mentor.

## Figures and Tables

**Table 1:** Parent participant responses by randomization assignment to parent mentor or community health worker regarding perceptions of the parent mentor or community health worker and their own confidence regarding their child's weight status. All scales with a possible range from 0 to 100, reported as mean (SD) using independent samples *t*-tests.

	Parent mentor participants (*n* = 23)	Community health worker participants (*n* = 23)	*p* value
Problem of childhood obesity in my community	77 (31)	81 (27)	0.67
Confidence in making healthier choices	97 (5)	99 (4)	0.39
Confidence in finding resources	97 (7)	97 (6)	0.93
Confidence that my child can achieve a healthy weight	94 (12)	96 (11)	0.65
Clarity of goals	96 (8)	93 (18)	0.47
Preparedness	94 (13)	94 (16)	0.98
Knowledgeable	94 (12)	91 (22)	0.62
Trustworthiness	95 (11)	94 (16)	0.96
Identify with presenter	96 (9)	81 (32)	0.04
Importance of the information presented	97 (7)	99 (2)	0.17
Confidence to apply what I learned	97 (7)	99 (3)	0.39
