# Supplementary material for: Factors Associated with Successful Mentoring of Parents Addressing Childhood Obesity: A Mixed Methods Approach
Source: Int J Pediatr. 2016 Nov 20;2016:5769621. doi: 10.1155/2016/5769621 (PMC5136387; doi:10.1155/2016/5769621)
Supplement: Supplementary file 1 — Supplemental file 1 contains the basic outline for the semi-structured interview conducted with each parent mentor. [file 5769621.f1.docx]

Interview questions:

1. Please tell me a little bit about your experience so far as a parent mentor.

2. Why did you choose to become a parent mentor?

3. How did you feel the first few times you met with a family?

a. Have those feelings changed at all?

b. Have you changed your approach to families since the beginning? Why?

4. Was there any part of the study that you feel uncomfortable with doing?

5. Please describe your most positive experience so far.

6. Please describe your most negative or challenging experience so far.

7. How have the community meetings been going so far?

8. What kind of support have you been offering to families that you mentor? What kind of help do you offer them?

9. What would you tell someone who wanted to be a parent mentor in the future?

10. How do you think families have received you as a mentor? How do you think they feel and how do you know? How do you tell them what your role is?

11. How have you used the parent mentor handbook that was part of the training, if at all?

12. How has this experience affected you? What have you taken away from the program so far?
